# Can pre-season fitness measures predict time to injury in varsity athletes?: a retrospective case control study

**DOI:** 10.1186/1758-2555-4-26

**Published:** 2012-07-23

**Authors:** Michael D Kennedy, Robyn Fischer, Kristine Fairbanks, Lauren Lefaivre, Lauren Vickery, Janelle Molzan, Eric Parent

**Affiliations:** 1 Faculty of Physical Education and Recreation, University of Alberta, Edmonton, AB, Canada; 2 Department of Physical Therapy, Faculty of Rehabilitation Medicine, University of Alberta, Edmonton, AB, Canada; 3 Glenrose Rehabilitation Hospital, Alberta Health Services, Edmonton, AB, Canada

**Keywords:** Exposure, Prognosis, Range of motion, Strength, Vertical jump, Sports injury

## Abstract

**Background:**

The ability to determine athletic performance in varsity athletes using preseason measures has been established. The ability of pre-season performance measures and athlete’s exposure to predict the incidence of injuries is unclear. Thus our purpose was to determine the ability of pre-season measures of athletic performance to predict time to injury in varsity athletes.

**Methods:**

Male and female varsity athletes competing in basketball, volleyball and ice hockey participated in this study. The main outcome measures were injury prevalence, time to injury (based on calculated exposure) and pre-season fitness measures as predictors of time to injury. Fitness measures were Apley’s range of motion, push-up, curl-ups, vertical jump, modified Illinois agility, and sit-and-reach. Cox regression models were used to identify which baseline fitness measures were predictors of time to injury.

**Results:**

Seventy-six percent of the athletes reported 1 or more injuries. Mean times to initial injury were significantly different for females and males (40.6% and 66.1% of the total season (*p < 0.05*), respectively). A significant univariate correlation was observed between push-up performance and time to injury (Pearson’s r = 0.332, *p < 0.01*). No preseason fitness measure impacted the hazard of injury. Regardless of sport, female athletes had significantly shorter time to injury than males (Hazard Ratio = 2.2, *p < 0.01*). Athletes playing volleyball had significantly shorter time to injury (Hazard Ratio = 4.2, *p < 0.01*) compared to those playing hockey or basketball.

**Conclusions:**

When accounting for exposure, gender, sport and fitness measures, prediction of time to injury was influenced most heavily by gender and sport.

## Background

Injuries among varsity athletes pose a significant challenge for both teams and individual players as demonstrated by surveillance of injuries in the National Collegiate Athletic Association (NCAA) [1-6]. A reportable injury is defined as one resulting from participation in an organized practice or competition and associated with restriction of participation or performance [1]. The opportunity for athletic injury increases with greater time spent participating [7]. Thus, measuring exposure to the possibility of injury in the greatest detail possible, such as minutes played in both practices and games is an important aspect of injury reporting [7]. Despite the fact that exposure is an important aspect of injury reporting, most studies report only on the occurrence of the injury and ignore quantifying exposure or the time from baseline evaluation to injury occurrence. The NCAA defines exposure as 1 athlete participating in 1 practice, which allows for a rate of injury to be determined (number of injuries / 1000 athlete exposures) [8], but does not account for the different exposure encountered by different athletes during practices and games.

Fitness evaluation and pre-participation evaluations (PPE; medicals) are standard practice in university sport [9]. The goal of these medical evaluations are to screen athletes for cardiac [10], musculoskeletal [11], and high-risk behaviour concerns [9]. Current standard for the musculoskeletal (MSK) part of the PPE include both medical history and a screening physical exam with history being very important for determining previous injuries [11]. Current evidence indicates that the efficacy of the physical screening exam to determine pathology and abnormalities or predict future injury is limited [11]. However, some tests commonly used to evaluate pre-season fitness may be prognostic factors for injury in varsity athletes [12,13].

Specifically, suboptimal musculoskeletal physical characteristics such as flexibility, muscle strength, muscle endurance, muscle power, and agility have been shown to be risk factors for injury in sports [1,13-18]. For example, flexibility is associated with an enhanced ability to dissipate and absorb forces and accommodate stress, thereby reducing injury [19] and strength/fatigue resistance [20] as well as agility, have been shown to prevent injury [21]. In contrast, vertical jump ability although beneficial to sport performance may increase risk of injury due to landing forces [22-24]. Thus, fitness and screening have been shown to be predictive of injury occurrence, however it is unclear whether pre-season fitness performance influences the time to injury.

Thus, the purpose of this study was to determine the utility of preseason fitness measures to predict risk of injury in varsity athletes when accounting for estimates of athlete’s exposure. We hypothesized that suboptimal preseason performance scores would be predictive of the time to injuries during the competitive varsity season.

## Methods

### Design

All participants (eligibility defined in participants) completed a battery of preseason fitness tests during the month preceding their competitive season starting. Exposure data was estimated retrospectively based on available data and expert assessment to determine the amount of time athletes were exposed to the possibility of injury in practice or competition. Injury data was obtained retrospectively using a self-administered injury report survey completed by participants at post-season team meetings for which attendance of all athletes (injured or not) was mandatory.

Testers were trained exercise specialists with experience conducting fitness testing according to accepted protocols [25]. A certified exercise physiologist with 10 years of experience in fitness testing was present at all test sessions to supervise the standard testing protocol. Fitness tests were administered in the same order specified as follows for all athletes to control for the effects of accumulating fatigue on a subsequent performance test: vertical jump, the sit and reach, the modified Illinois agility, push ups, curl ups and the Apley’s range of motion test. Standardized guidelines were followed in the administration of all the tests [21,25,26]. All fitness tests included have shown adequate reliability in order to draw inferences on individuals (ICC > 0.9) [21,27-30].

### Participants

The sample consisted of 6 varsity teams including men’s hockey (n = 23), women’s hockey (n = 18), men’s volleyball (n = 14), women’s volleyball (n = 10), men’s basketball (n = 9) and women’s basketball (n = 12). This sample of varsity teams includes sports where sport participation involves use of both the upper and lower extremities where both genders are involved. Descriptive data is presented in Table 1. All 86 participants provided written informed consent to participate in accordance with guidelines from the Research Ethics Board for the Faculty in the spirit of the Helsinki declaration. To be eligible for inclusion, an athlete must have participated in a varsity game or practice during the 2008-2009 season and have data for both the preseason fitness testing and the post-season injury survey. At the time of the pre-season fitness testing, all athletes were medically cleared to participate as determined by their Pre-Participation Evaluation (PPE) [31]. Each PPE was completed by the athlete and referring physician, then submitted to the head therapist for review. The head therapist then either cleared each athlete for participation (no flagged issues on PPE) or provided arrangements for additional referrals or evaluations as flagged by specific identified issues on the PPE. Only when subsequent referrals and evaluations were completed in those athletes who required follow up did the head therapist clear that athlete for participation.

**Table 1:** Descriptive variables of each team (mean ± SD)

**Team**	**Age (yrs)**	**Height (cm)**	**Weight (kg)**	**Body Mass Index (BMI)**
Men’s Basketball	20.6 ± 1.4	189.9 ± 7.0	91.3 ± 12.1	25.2 ± 2.4
Men’s Hockey	22.9 ± 1.4	183.1 ± 4.0	85.9 ± 7.2	25.6 ± 1.6
Men’s Volleyball	20.1 ± 1.1	195.9 ± 7.0	90.2 ± 8.8	23.5 ± 1.6
Women’s Basketball	20.0 ± 1.9	181.1 ± 6.8	74.3 ± 8.5	22.6 ± 2.0
Women’s Hockey	20.0 ± 1.5	170.0 ± 12.1	69.5 ± 6.6	24.4 ± 4.4
Women’s Volleyball	20.3 ± 1.8	180.7 ± 6.7	69.3 ± 8.8	21.2 ± 2.0

### Procedures

#### Preseason fitness measures

The vertical jump test estimated instantaneous anaerobic power and lower body strength. No run-up, step up, or pre-jump was permitted. One familiarization jump was allowed to ensure the participant executed the correct technique. Three testing trials were performed with a rest period of 10-15 seconds between each trial. A vertec device was used to measure jump height. The trial with the greatest jump height was recorded to the nearest 0.5 inch. Maximal jump height was converted to centimetres and the difference between the maximum jump height and an athlete’s standing reach height was recorded as the vertical jump height (cm).

The sit and reach test was used to evaluate lower back and hip flexibility [32]. Participants were encouraged to stretch hamstrings prior to testing although no formal warm-up was administered. Participants were asked to sit with their legs fully extended, the ankle at 90° and to reach forward as far as possible with their outstretched arms and fingers by bending their back and hips. The maximal position of flexion had to be held for 2 seconds and 2 trials were performed with a 10-15 second rest between. The maximum sit and reach distance was recorded to the nearest 0.5 cm.

The modified Illinois agility test evaluated the participants’ ability to accelerate, decelerate, turn in different directions, and run at different angles [26]. The test was administered as described by Vescovi et al [21],. where four cones were used to mark the rectangular agility area (10 meters long, 5 meters wide). Another 4 cones were placed inside the centre of the area at a distance of 3.3 m apart. Participants began on their own time and the tester started the stopwatch at the participant’s initial movement. Participants ran straight to the second cone, zigzagged through the series of cones in the centre and finished by running around the final 2 cones of the course. Time was stopped when the participant crossed the finish line. Two trials were permitted with a 1-2 minute rest period in between and the best time was recorded with a handheld stopwatch to the closest hundredth of a second.

The push up test was used to evaluate upper-body endurance specifically, pectoralis major, anterior deltoids and triceps [32]. No pause was allowed at elbow extension and a self-selected tempo had to be maintained throughout the test. The number of repetitions were counted until fatigue or until the participants were unable to maintain proper technique over 2 consecutive repetitions.

The curl-up test evaluated the endurance of the abdominal musculature that provides a foundation for trunk and spine stability [33]. The test was administered to a metronome beat set at 50 beats per minute (25 curl-ups per minute). One warning about improper form was permitted prior to final termination of the test. The number of curl-ups that met the proper technique criteria was recorded [25]. The partial curl-up test was used because it produces the highest activation of the abdominal musculature (rectus abdominus, external obliques, and lower abdominal stabilizers) with minimal activation of the hip flexors [34].

The Apley’s ROM test assessed shoulder flexibility, specifically medial rotation with adduction and lateral rotation with abduction. The test reflects functional capacity at the shoulder [35]. Participants were required to remove their shirt to bare skin or bra top. The tester held a measuring stick (cm) with the 20 cm mark at the level of the inferior angle of the scapula. Participants were instructed to reach into lateral rotation and abduction with palm facing anterior. Second, the participants were asked to reach into medial rotation and adduction with palm facing posterior. Participants repeated the same motions on the other side. Measurements were recorded as a positive (good range) or negative (limited range) value measured from the level of the inferior angle (20 cm mark) to the level of the 3^rd^ digit. Measurements were recorded to the nearest 0.5 cm.

#### Exposure estimate

Exposure was quantified as the total amount of time participants were exposed to the possibility of injury in practice or competition prior to injury.

Practice exposure time was determined by totalling the amount of team practice time (in minutes) that individual athletes participated in prior to the occurrence of their first injury. Team practice time was calculated using each team’s practice schedule throughout the season. Due to the retrospective nature of this analysis and because practices are mandatory, the estimate of practice exposure is based on the assumption that athlete’s were exposed in the full duration of scheduled practice up until the day on which injury occurred. The absence from practice due illness or personal reasons was not documented.

Based on the retrospective nature of game exposure estimations, game exposure was determined differently for each sport using the best available data. Individual estimates of game exposure were calculated. This was to account for the significant differences in playing time that a starter or first line player might have compared to a substitute or fourth line player. For ice hockey teams, game exposure was derived from coach-estimated average playing time per game per athlete at the end of the competitive season. Using this estimation, total game exposure time per participant was calculated up to the date of injury using the team game schedule. Game exposure time for basketball players was taken directly from game statistics, which recorded the number of minutes each individual played per game. For volleyball players, game exposure was calculated by multiplying the number of sets each athlete played per game by the number of rallies within those sets, their team’s average rally time, and a coach-estimated average percentage of court time per set that each athlete played over the season. The lattermost variable accounts for differences in court time not recorded by game statistics. Set played and numbers of rallies were retrieved from game statistics. Average rally time per team was determined by reviewing 5 rallies from each of 3 randomly chosen videotaped games from both the men’s and women’s 2008-2009 season games.

#### Injury surveillance survey

The survey was modified from the National Collegiate Athletic Association (NCAA) Injury Surveillance System [8] to be completed by the athletes and for data capture using the Cardiff Teleform system (New England Survey Systems, Brooklyn, MA). The survey was simplified and re-formatted so that participants may effectively report injuries retrospectively. Face validity of the survey was assessed with 2 men’s league soccer teams and 2 women’s recreational basketball team. Feedback on readability and time to complete survey was collected and appropriate changes were made. Injury data was not retrieved from the central care training room athlete’s records. This is because central care only records injuries of those athletes who make a formal appointment to see an athletic therapist. Using only central care data we could have missed injuries that were treated outside of central care. Our strategy is supported by recent research showing that a self-report internet survey of injury reporting by parents of adolescent soccer players captured more injuries than athletic therapist reported injuries [36]. In addition, calendars of each team’s pre-season and competitive season were distributed at their respective meetings to help athletes determine the exact date at which the injury occurred. Furthermore, trainers were present to assist athletes in recalling injuries that occurred, the type of injury and when the injury occurred.

### Statistical analyses

Descriptive analysis consisted of means and standard deviations for subject preseason performance. Frequencies were estimated for each type and location of injury. Univariate analysis of the ability of the preseason measures to predict time without injury was completed using Pearson Correlation coefficients.

Proportional-hazard (or Cox) multivariate regression analysis was used to determine the predictive ability of each pre-season measures for the hazard of injury while taking into account gender and sport. The primary dependent variable in this study was time to initial injury or to the end of the season for athletes who were not injured. Time to initial injury was reported as the percentage of exposure in games and practices from the total season duration prior to injury. For athletes who were not injured, 100% exposure was assumed. Baseline or time zero was determined as the first official practice for each team. The independent variables in this study were the 5 preseason baseline measures (vertical jump, sit and reach, Apley’s ROM test, push up test and modified Illinois agility test). Hazard is defined as the probability of being injured at a given point in time, having remained uninjured up until that point [37]. Cox regression accounts for different amounts of exposure to injury amongst participants. It enables the difference between survival times (or time remaining injury free) of particular groups of participants to be predicted while allowing for other prognostic factors or covariates to be considered at the same time [38]. The preseason scores were entered into the model as continuous variables. Upper and lower extremity range of motion data was entered in the analysis as a distance from the mean to reflect the hypothesis that too much or too little ROM was hypothesized to be a risk for injury. Hazard ratios were reported with 95% confidence intervals (95% CI). Statistical analysis was completed using SPSS v15.0.1.1 (SPSS) using an alpha level of 0.05.

## Results

### Participant description and injury survey

A total of 46 male and 40 female athletes completed preseason baseline measures (Table 2) and the post-season injury survey. Seventy six percent of athlete’s reported one or more injuries during the 2008-2009 season (Table 3), over half of which were new injuries (63.1%). Twenty nine percent were recurrence of past injuries. Injuries to the lower extremity were the most common with 33.8% of athletes reporting injury to this area. The most common injury type reported was incomplete muscle or tendon strain (23.1%).

**Table 2:** Mean and standard deviation for preseason baseline measures

**Preseason Baseline Measure**	**N**	**Mean**	**Standard Deviation**
Upper extremity right internal rotation	86	7.80*	6.63
Upper extremity left internal rotation	85	11.37*	5.82
Upper extremity right external rotation	86	−8.24*	3.80
Upper extremity left external rotation	85	−8.29*	3.30
Upper extremity strength (push up)	85	22.44‡	12.45
Lower extremity range of motion (sit and reach)	85	38.68*	7.95
Lower extremity power (vertical jump)	83	53.94*	10.34
Modified Illinois agility	60	11.22†	.70
Curl ups	85	28.22§	16.00

* Indicates measured and recorded in cm.

† Indicates measured and recorded in seconds.

‡ Indicates the number of push ups performed.

§ Indicates the number of curl ups performed.

**Table 3:** Frequency of body part injured and injury types

**Body Part**	**Injury Type**											**Total**
	**Bruise**	**Tendinitis**	**Ligament sprain incomplete**	**Ligament sprain complete**	**Muscle or tendon strain incomplete**	**Dislocation partial**	**Dislocation complete**	**Fracture**	**Stress fracture**	**Herniation**	**Other**	
Upper extremity	3	3	8	1	1	1	2	1	0	0	0	20
Lower extremity	1	4	5	0	6	0	1	0	1	0	4	22
Upper back/ neck/ ribs	2	0	0	0	4	0	0	0	0	0	0	6
Lower back/ pelvis	0	1	0	0	4	1	0	0	0	2	3	11
Head	1	0	0	0	0	0	0	0	0	0	5	6
Total	7	8	13	1	15	2	3	1	1	2	12	65

Most injuries occurred during the regular season (61.5%); 33.8% occurred during the preseason, and 4.6% during the post season (Table 4). The majority of athletes did not miss any games due to their injury (73.8%); however most missed 1 or more practices due to their injury (55.4%). All but 1 athlete sought some form of medical attention following injury (98.5%).

**Table 4:** Number of injuries to occur in pre season, regular season and post season play

**Team**	**Practice**			**Game**		
	**Pre season**	**Regular Season**	**Post Season**	**Pre season**	**Regular Season**	**Post Season**
Female hockey	5	3	0	0	7	0
Female volleyball	3	4	0	3	0	0
Female basketball	3	5	0	0	1	0
Male hockey	0	2	0	2	6	3
Male volleyball	4	8	0	0	1	0
Male basketball	1	0	0	1	3	0
Total	16	22	0	6	18	3
Total full season	38			27		

Injuries occurred in a greater percentage of games (11.8%) than practices (6.9%). Over half of practice injuries occurred during regular season practice (57.9%). The remainder occurred in preseason practice (42.1%). No injuries occurred in post-season practice. Sixty-six percent of game injuries occurred during regular season competition (66.6%). Twenty two percent occurred in pre season games (22.2%), while 11.1% occurred in post-season games (Table 4).

### Time to injury in varsity athletes by gender and teams

**Figure 1:**
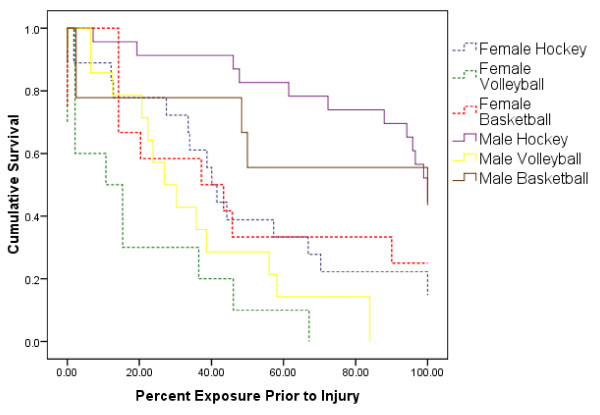
Kaplan Meier survival curves of the time to initial injury for each varsity team.

Mean times to initial injury were significantly shorter for females (40.6% of total season) than for male athletes (66.1%). Mean times to initial injury reported in percent of the total varsity participation were significantly shorter for volleyball players (27.4%) than for hockey players (68.5%) or basketball players 57.6% (Table 5). Kaplan Meier survival curves for each team showing the percent athlete not injured at different percentage of the total varsity participation are presented in Figure 1. Female and male volleyball players had the shortest mean time to injury (19.3% and 35.7%), followed by women’s hockey (48.9%) and women’s basketball (45.9%). Men’s basketball had a mean time to initial injury of 66.8%. Male hockey players had the longest mean time to initial injury at 83.8%.

**Table 5:** Mean time to initial injury expressed as % of season completed with 95% confidence interval

	**Mean Time to Initial Injury (%)**	**95% Confidence Interval (Lower limit; Upper limit)**
Team:		
Female hockey	48.9	33.1-64.8
Female volleyball	19.3	5.0-33.7
Female basketball	45.9	23.5-68.3
Male hockey	83.8	72.2-95.4
Male volleyball	35.7	21.9-49.4
Male basketball	66.8	37.2-96.3
Gender:		
Female	40.6	29.5-51.6
Male	66.1	55.3-76.8
Sport:		
Hockey	68.5	57.7-79.3
Volleyball	27.4	17.4-37.3
Basketball	57.6	39.2-76.0

### Correlations between preseason test scores and time to injury

Only 1 significant univariate correlation was observed between preseason test scores and time to injury (Table 6). Weaker upper extremity strength (push ups) was associated with shorter time to injury (Pearson’s r = 0.33, *p < 0.01*).

**Table 6:** Pearson correlation of preseason baseline measures and time to initial injury expressed as % of season completed before injury

**Preseason Baseline Measure**	**N**	**Pearson Correlation**	***p***
Right internal rotation deviation from mean	86	−0.14	0.22
Left internal rotation deviation from mean	85	0.07	0.52
Right external rotation deviation from mean	86	0.03	0.76
Left external rotation deviation from mean	85	0.09	0.42
Upper extremity strength (push ups)	85	0.33	<0.01
Lower extremity range of motion deviation from mean (sit and reach)	85	0.15	0.16
Lower extremity power (vertical jump)	83	0.13	0.23
Illinois agility	60	−0.11	0.39
Curl ups	85	−0.08	0.49

### Multivariate prediction of time to injury

Proportional-hazard (Cox) regression was used to determine the effect of each independent variable on the hazard of injury. Cox regression analysis including gender and sport as covariates revealed that none of preseason measures of strength, range of motion, and agility significantly impacted the hazard of injury. Only, vertical jump performance showed a trend toward impacting injury hazard. As vertical jump scores increase, so does the hazard of injury (HR 1.04, *p < 0.10*). The Cox regression model predicting time to injury using only gender and team as predictors is reported in Table 7. Regardless of sport, female athletes had significantly shorter time to injury than males (HR = 2.2, *p < 0.01*). Athletes playing volleyball had significantly shorter time to injury than either basketball or hockey (HR = 4.2, *p < 0.01*).

**Table 7:** Cox regression with gender and team acting as covariates

**Covariate**	**Hazard ratio (95% Confidence Interval)**	***p***
Gender		
Female	2.21 (1.34-3.64)	<0.01
Male	1	
Sport		
Volleyball	4.25 (2.05-8.79)	<0.01
Hockey	1.08 (0.55-2.09)	0.83
Basketball	1	

## Discussion

### Main findings

This study sought to develop a predictive model for injury in varsity athletes from sports where both upper and lower extremities are used using preseason performance measures. The study accounted for estimated exposure to the risk of injury for male and female basketball, volleyball, and hockey athletes. Overall, the results indicated that female gender is the most predictive factor in determining time to injury in game or practice regardless of preseason performance. Further, volleyball had significantly shorter time to injury than the other sports studied.

Univariate associations suggested that athletes with limited upper extremity endurance as demonstrated by low push up performance had a shorter time to injury. However, when accounting for the relationship between gender and push up performance in the multivariate model, female gender better predicts shorter time to injury. Similarly, Augustsson et al found that males performed significantly more push ups than females and had 44% greater upper body endurance strength [39]. They suggested that females who train upper body endurance may be more likely to avoid injury [39]. In this study, pushups data alone was not sufficient to predict time to injury. However, our results provide some support to investigate the belief that strength and conditioning in athletes may be a good prevention strategy not only for occurrence but also for time to injury.

Vertical jump was the only other pre-season performance measure showing potential to predict time to injury. Athletes who had higher vertical jump scores showed a trend toward being injured earlier. This finding may appear counterintuitive given that greater muscular or aerobic performance scores are typically desirable in sports. However, Martel et al claim that plyometric training of high intensity and high impact to improve vertical jump height increases the possibility for muscle damage and injury [24]. Additionally, Hewett et al identified female athletes with a higher vertical jump performance scores as having higher risk landing profiles and that high jumping females were more likely to incur injuries, especially at the knee [22]. In volleyball and basketball high vertical jumps are desirable, however this research and those previously mentioned [22-24] suggest that this greater performance attribute may be associated with increased risk of injury. Additionally, athletes with the greatest vertical jump height are likely to play more games and thus have greater exposure to injury in competitive situations. Therefore the combination of increased performance and exposure may put athletes in starting positions at higher risk of injury compared to other players.

Of the injuries collected over the 2008/2009 varsity season, injuries occurred in a greater percentage of games (11.8%) than practices (6.9%). This finding is consistent with data from the NCAA where athletes were 3.5 times more likely to sustain an injury during a game than practice [1]. Both our study and the NCAA study found that injuries occurring in games were greatest for athletes involved in contact sports, compared to sports traditionally considered “non-contact” such as volleyball [2]. Contacts are more frequent in game situations [1].

In the present study, lower extremity injury was more prevalent than upper extremity injury in hockey, volleyball, and basketball which is consistent with previous findings [40-43]. The most common types of injuries in the present study across all sports examined were muscle strain (23%), ligament sprain (20%), and tendonitis (12%). These findings are consistent with other studies reporting muscle sprain/strain as the most prevalent injury [41-43]. However, Hootman et al report ligament sprain (14.8%) as the highest injury prevalence [1]. This discrepancy may be due to the difference in injury definition used as Hootman et al [1] only accounted for injuries that resulted in limitation of participation in competition or practice, while we defined injury as one that could have resulted in limited participation, or in the athlete seeking medical attention. In our study, we may have captured more injuries as some participants may have incurred an injury and sought medical treatment but did not restrict themselves from practice.

#### Gender and sport differences

Our study found that females were more likely to be injured and had a shorter mean time to injury than males. These results are consistent to the majority of studies found in the literature [13,39,44-46]. For example Murphy et al found a discrepancy between male and female injury incidence among diverse populations [13]. They found females to be at greater risk than males when identifying risk factors for lower extremity injury [13]. On the other hand others have found that gender was not an important factor in sport injuries for athletes involved in volleyball, basketball, soccer, wrestling and running [40]. One reason for this difference may be because men are less likely to report injuries [47]. The increased likelihood to report injuries by females may be a cultural bias that may be considered in future studies relying on self-reporting of injuries. For our results, this bias if true likely influenced only injury prevalence reporting and not time to injury in this study.

To our knowledge, no previous studies examined gender differences with respect to mean time to injury thus our finding that female’s time to injury was shorter than males is novel. We are confident in the finding that mean time to injury is shorter in females because although our exposure estimate may not be exact the error in estimating exposure in our study should be the same for male and females. Others have identified other female risk factors for increased risk of injury.

Our findings demonstrate that both men’s and women’s volleyball had the highest rate of injury and the shortest time to injury. Compared to the longitudinal NCAA data our findings are less than the NCAA women’ volleyball data which reported 4 injuries per 1000 athlete exposures. The differences might exist due sample size and further longitudinal data collection with Canadian varsity athletes might elucidate any real differences compared to the NCAA data. However, it is noteworthy that we cannot technically compare exposure differences because the NCAA exposure data is a frequency measure defined as 1 athlete participating in 1 practice [8], whereas our exposure is a calculated estimate of exposure in minutes. It has been indicated that a limitation of the NCAA data is the sensitivity of the exposure estimate whereby, **“**future authors should use a finer level of exposure measurement, such as player-minutes” [1] In this case our estimate of exposure in minutes allows for time to injury to be investigated, with the finding that volleyball has significantly shorter time to injury than basketball or hockey.

### Limitations and strengths

Accounting for and documenting practice and game exposure when predicting injury is a novel aspect of this study. The collection of the measurement of the potential predictors of injury was collected before the exposure. However, injury information was collected retrospectively with a risk of recall bias. For feasibility reason, we based the injury data survey on the NCAA Injury Surveillance System modified so that an athlete could complete the survey retrospectively. A member of the research team was present to answer questions or clarify items when the survey was administered. Furthermore, practice and competition schedules were used to help athletes remember injury dates and to reduce any recall bias that may have occurred. Others have shown that retrospective tracking of injuries is sufficiently accurate in athletes [48]. Our injury survey also ensured that we captured all injuries including those that may not have been reported to athletic therapy staff in a prospective injury tracking system because we were asked the athletes themselves.

We combined upper and lower extremity injuries when examining the relationship between preseason performance measures and injury occurrence. This may have reduced the predictive power of the preseason performance measures used in this study because some preseason measures may only be predictive of specific injuries. However, the intent was to assess the ability of the preseason measures to identify athletes at risk of any injuries in sports involving both upper and lower extremity activities.

Missing data may have influenced the results. Due to a scheduling conflict men’s hockey did not complete the Modified Illinois Agility test. Other missing fitness measures (<3 per measure) were due to either scheduling conflict or exclusion of invalid result due to instructions not being followed.

Future studies should continue to utilize a comprehensive approach to document time exposed to injury. Larger sample sizes would help confirm if preseason measures are significant predictors of a specific injury in multivariate models when still accounting for gender and sport. Alternatively, because we found differences between genders and between sports, we recommend focussing on only one gender and one sport when planning studies on predictions of shorter time to injuries. Males and females practicing different sports may have different backgrounds in terms of training, and previous injuries. Further, different sports include different activities. Poor scores on fitness measures may predict shorter time to injuries when exposed to risky activities more prevalent in a given sport than another. Nevertheless, our results show that the preseason measures selected in the present study were not strong predictors of short time to injuries over and above gender and sport in sports that were selected because they involved both upper and lower extremity use. Our results provide novel evidence that gender and sports are key predictors of time to injury in out selected varsity sports. To our knowledge this information was lacking from the literature as analyses predicting time to injury are rare in the varsity sport literature.

Likely this means that a different predictor set will be required for different sport even though for simplicity varsity programs may have benefited from using a common dataset for all sports and genders.

In the future, it may be beneficial to examine the predictive ability of more performance measures, however, to ensure the feasibility of preseason screening the added measures should have the ability to be administered rapidly, and require little equipment. Identifying more performance measures with the ability to predict injury is of utmost value for varsity athletics. In the future, collecting injury information and documenting exposure in minutes prospectively should be considered. Future research should consider the impact of other conditions that may influence participation in practice or games when estimating exposure. Also, the modified varsity sport injury survey could be compared to prospective injury recording in order to confirm its validity.

## Conclusions

Our study found the prevalence of injury types and location to be similar to those found in other studies. When accounting for exposure, gender and sport, performance measures were not found to be significant predictors of time to injury. Time to injury was influenced most heavily by gender and sport. Our study did not support the hypothesis that baseline performance measures would predict time to injury.

## Misc

Michael D Kennedy, Robyn Fischer, Kristine Fairbanks, Lauren Lefaivre, Lauren Vickery, Janelle Molzan, Eric Parent contributed equally.

## Competing interests

The author(s) declare that they have no competing interests.

## Author contributions

MDK contributed to the design, analysis and drafting the manuscript; RF contributed to the design, created the injury surveillance instrument, analyzed and wrote results and helped draft manuscript; KF contributed to the overall design, data collection and manuscript preparation; LL assisted with the conception of the project, collected data and oversaw the development of the methods; LV assisted with the overall design, oversaw data collection and assisted in manuscript preparation; JM assisted in overall design and development of the methods and exposure data collection; EP oversaw the design development, the statistical analysis and assisted in the drafting of the manuscript. All authors read and approved the final manuscript.
